# Detection of Corporate Environmental Information Disclosure Falsification Based on Support Vector Machine

**DOI:** 10.1155/2022/5270963

**Published:** 2022-08-16

**Authors:** Yinwen Li, Xiang Cai, Huaping Sun, Xingxing He

**Affiliations:** ^1^School of Business, Beijing Technology and Business University, Beijing 100048, China; ^2^School of Business, Guilin University of Electronic Technology, Guilin 541004, China; ^3^School of Economics and Management, Hebei University of Technology, Tianjin 300401, China; ^4^School of Finance and Economics, Jiangsu University, Zhenjiang 212013, China

## Abstract

Environmental information disclosure (EID) is an important embodiment of corporate social responsibility. With the awakening of public awareness of environmental protection and the increasing pressure of environmental preservation, enterprises tend to strategically manipulate environmental information for the pursuit of profit, which will consequently lead to environmental information disclosure falsification (EIDF) and disruption of both the market regulatory order and the development of green economy. In this article, support vector machine (SVM) technique is applied to construct the detection model of corporate EIDF. Based on the theory of “public pressure,” the detection indicators will be improved from three aspects: public pressure, corporate governance, and financial indicators. The training set and test set are constructed by combining the manually collected cases of environmental administrative penalties from 2015 to 2019 with the indicator information of nonfinancial listed enterprises in China's A-share market, and the SVM detection performance is compared with the logistic regression of the benchmark model. To solve the problem of category imbalance, we have introduced the Borderline-SMOTE oversampling technique. Based on the detection results of SVM and Borderline-SMOTE, we find that the Borderline-SMOTE-SVM model has the best detection performance, surpassing the SVM and logistic regression models. These conclusions have constructive policy implications for regulatory agencies, investors, the third-party service sector, enterprises, and government policy-making to achieve high-quality corporate EID.

## 1. Introduction

In recent years, with the acceleration of industrialization and urbanization, the increasingly prominent environmental problems have become an obstacle to sustainable development. Traditional financial reports cannot satisfy the needs of information users to understand companies' environmental problems, and the disadvantages of accounting information disclosure are becoming progressively obvious. Under such circumstances, enterprises are prompted to disclose environmental information, such as environmental reports, sustainable development reports, social responsibility reports, and other nonfinancial reports [1]. High-quality EID can enhance corporate reputation [2], improve financial performance and financing capacity, and reduce credit costs [3]. In addition, it can also urge enterprises to better fulfill their environmental responsibilities and reduce environmental pollution [4].

However, EID also has costs [5], because the environmental information disclosed by companies will be subject to strict review by regulators, which will bring them pressure to solve environmental problems [6]. In addition, EID may become the basis for enterprise environmental risk assessment, and investors prefer companies with good environmental performance. Taking those factors into consideration, enterprises will adopt selective EID.

In China, EID is still in its infancy. Most of the enterprises' EID is mainly qualitative description, with little quantitative information, incomplete content, and low transparency [7]. Due to the imperfect internal and external supervision mechanism, enterprises often have the space to select the content of EID. In addition, China has not yet formed a complete and unified environmental accounting system. Many listed enterprises in China have been trying to whitewash their corporate image recently to sheerly pursue profitability, thus giving rise to frequent occurrence of EIDF, such as the “greenwashing” of ESG report and the manipulation of sustainability report. For example, the 2019 Annual Report Environmental Information Disclosure Inspection Report for Listed Enterprises has shown that among more than 3,600 listed enterprises most just kept a relatively reticent attitude towards “environmental fines” and refused to disclose environmental penalties. Similarly, a 2017 research report by the Center for Environmental Economics at Fudan University pointed out that nearly half of the sample enterprises often avoided direct answers in key places, even if they took the initiative to disclose environmental information. For instance, when it comes to the specific discharges of pollutants, enterprises are likely to use ambiguous expressions such as “striving to implement energy conservation and emission reduction targets,” but it is impossible to know the key information as what measures have been taken and how many pollutants have actually been reduced. In addition, many enterprises' information disclosure is selective, reporting good news only and avoiding bad news. As for positive news, they release it in an optimistic and comprehensive way, but for negative news such as receiving penalties, they often choose to conceal the fact [8]. Those enterprises strategically “rinse” environmental information by means of concealment, misrepresentation, and evasiveness, which has become a chronic disease, which will mislead investors, spoil market order, and pose a huge threat to environmental governance [9]. Therefore, it is necessary to identify the EIDF.

The concealment of EIDF, coupled with the huge economic benefits and the low cost of “greenwashing,” makes this misbehavior difficult to detect. It simultaneously indicates that traditional identification methods could not play an effective role. With the advent of the era of big data, machine learning methods that developed in the 20th century have significant advantages in efficiently acquiring knowledge, processing complex data, and building high-precision models. The main motive of this study is to utilize machine learning methods to help people extract effective information from a large amount of complex data, so as to find the law of EIDF and accurately identify their behavior of falsification.

Currently, the existing literature has performed preliminary research on the motivation and identification of corporate EIDF, but it is comparatively weak. Huang summarized the external and internal factors of enterprises' “greenwashing” of ESG report. The external factors included exploiting the flaws of institutional arrangements, catering to the preferences of rating agencies, meeting the needs of green financing, and improving the environmental image of enterprises. The internal factors included inadequate governance mechanisms, incompetent internal controls, insufficient database, and the poor ethical atmosphere [10]. The empirical analysis of He and Ren confirmed the existence of manipulation of EIDF, and it has a left-skewed “inverted L” trend. By constructing a “public pressure bundle” measurement model, the PSM method and the binary logit model were adopted to test the validity of the measurement model for identifying falsification [11]. Based on the existing literature, this research will utilize the “public pressure” theory to perfect the index system and identify EIDF through machine learning methods.

Due to the inefficiency of EID, how to achieve effective detection with limited information is the focus of this research. Therefore, machine learning methods are introduced for better detection and identification. Currently, there are three main types of machine learning: supervised learning, unsupervised learning, and reinforcement learning. Since the categories of our sample data are known, we mainly use classifiers in supervised learning to identify falsification. Commonly used classifiers include logistic regression, support vector machine, Bayesian classification, decision tree, random forest, and artificial neural network. Among a number of algorithms, logistic regression is the classical machine learning algorithm, which is widely used for the reason that it is easy to understand, simple to operate, and fast to run, while SVM has excellent ability to generalize and distinguish, which is very popular in recent years. Considering everything, this study mainly uses SVM to construct a model and compares the results with those of logistic regression (LR) models to verify the validity of SVM. Given the category imbalance caused by the small number of falsified positive samples, we combine the Borderline-SMOTE method to optimize the model and make the results more reliable.

The marginal contributions of this study are as follows:

1. Based on the “public pressure” theory, we explore the motives of EIDF and construct the indicator system from three perspectives: public pressure, corporate governance, and financial indicators. It is found that public pressure on enterprises mainly comes from the government, investors, and media. Corporate governance mainly includes internal supervision and equity structure, which can be summarized as the size of supervisory board, equity concentration, and equity balance. Financial indicators include profitability, solvency, and growth capacity.
2. There are now few studies on the detection and identification of corporate EIDF, and most of the existing literature adopts traditional econometric and statistical methods such as least squares regression (OLS). These methods mostly provide linear models, which could be inadequate when dealing with high-dimensional, complex empirical data. Hence, this study introduces the SVM method to fill this gap and demonstrates its effectiveness and robustness in falsification detection, which will provide more research perspectives on EIDF behavior.
3. The concealment of falsification has made the categories of corporate EIDF samples extremely unbalanced. Consequently, the training model tends to focus more on the categories with a high number and “downplay” the minority classes, thus adversely affecting the model's generalization ability in the test data. For the minority classes, it was found that Borderline-SMOTE has better TP rates and F-values than SMOTE and random oversampling methods [12]. Therefore, we creatively introduce this method to synthesize a few samples artificially, thus improving the reliability of the model. As far as we know, this method has been widely used in other discipline areas, but is seldom used in accounting disclosure. Thereby, this innovative research method ensures that the research result is more convincing.

The remainder of this study is organized as follows: Section 2 summarizes the motivation of enterprise EIDF, the application of machine learning in economics and management, and the improvement method of class-imbalance problem.  Section 3 introduces our research methodology, including a detailed description of the indicator system construction, modeling methods, and performance assessment methods.  Section 4 describes the data selection, data sources, and the mean characteristics of the sample.  Section 5 gives the empirical results and provides details of the empirical study.  Section 6 offers a conclusion and analyzes the limitations of this study.

## 2. Related Work

In March 1989, the seventh session of the United Nations Intergovernmental Working Group of Experts on International Standards of Accounting and Reporting first discussed the issue of EID [13]. In 1998, the 15th meeting of the United Nations Intergovernmental Working Group of Experts on International Standards of Accounting and Reporting discussed and adopted the Position Announcement on Environmental Accounting and Reporting. This is the first systematic and complete international guide on environmental accounting and reporting in the world. Since then, the Chinese government and relevant institutions have issued a series of norms related to EID, and academic world has also conducted a lot of research on EID. Many scholars believed that the EID of the listed enterprises in China had low quality and practicability, and they have low transparency for the lack of third-party audits, so their credibility was questionable [14]. By studying the environmental protection inspection carried out by the Ministry of Environmental Protection in 2009, Hu found that there were serious inconsistencies in the content, tone, and format of the environmental performance information disclosed by listed enterprises [15]. Based on the social responsibility reports of China's power enterprises, He and Zhu found that enterprises tended to disclose positive information and descriptive information, which was difficult to verify, but seldom disclosed negative information [16]. Huang made a judgment on the “greenwashing” behavior of corporate ESG reports, and defined “greenwashing” as the behavior of enterprises and financial institutions exaggerating the efforts and effectiveness of environmental protection issues, and making overstatements about environmental protection and resource use in ESG reports or sustainability reports [10]. Wang and Ding pointed out that environmental information was significantly different from other information, and its diversity and complexity determined that enterprises would have greater disclosure autonomy and space for manipulation [17]. All the above literature shows that corporate environmental information falsification does exist, and it presents different forms with the development of China's EID system.

For the analysis of the motives of corporate EIDF, the existing literature are still weak. Most studies are based on legitimacy theory [18, 19] and stakeholder theory, and focus on factors affecting the quality of EID such as government regulation, media attention, and corporate characteristics [20]. For media regulation, Brown and Deegan found that the quality of EID in annual reports was significantly correlated with media attention [21]. For the characteristics of enterprises, Meng, Zeng, Tam, and Xu took 782 A-share listed enterprises in China's manufacturing industry from 2006 to 2008 as the research object and found that there was a significant statistical relationship between corporate governance structure and EID level. They came to the conclusion that improving corporate governance institutions would help improve the level of EID [22]. Furthermore, based on an empirical analysis of listed manufacturing enterprises in China, Wang concluded that public pressure and brand reputation are the important factors influencing corporate EID, and the influence of internal governance factors is relatively weak [23]. Additionally, enterprises with high equity concentration have a correspondently higher level of information disclosure. Plumlee and other scholars stated that there is a correlation between firm value and the quality of voluntary EID [24]. Shen, Huang, and Guo believed that enterprises with poor environmental performance face greater political pressure and legal threats, and would try to “defend” themselves with optional disclosure. And they found that there is a negative correlation between environmental performance and EID [25]. He and Ren put forward the “public pressure bundle” model with reference to the idea of earnings manipulation measurement [26] and believed that the generation of corporate falsification was the result of the interplay of the inhibitory effect of “external pressure bundle” and the inducing effect of “internal pressure bundle” [11]. For example, the increase of external pressure such as legal regulation and public opinion pressure will make enterprises afraid of the legal system and supervision, thus weakening speculative behavior and making falsification less likely to occur, while the increase of internal pressure such as increased debt servicing pressure and reduced growth capacity will make enterprises compete for scarce resources and government subsidies through falsification. Based on the above literature, our study analyze what kind of enterprises are prone to EIDF. The difference lies in that we have carefully distinguished the indicators, improved the basic indicator system from three perspectives—public pressure, corporate governance, and financial indicators, and creatively introduced the size of the supervisory board and ISO certification to reflect the level of internal supervision and social reputation of enterprises.

Recently, machine learning has attracted the attention of economists. Huber [27] applies machine learning methods to the selection of hedge funds to achieve stable portfolio performance, which can help identify the similarities in return structures of hedge fund managers, and hence avoid concentrations in a portfolio. Gu, Kelly, and Xiu found that machine learning methods can be used to select effective variables from a large number of existing variables for stock return prediction [28]. In terms of classifier selection, SVM has grown significantly with its excellent learning performance in fields of pattern recognition and regression analysis, and has been widely used in image recognition, text recognition, face detection, gene classification [29–31], and so on. Jones, Johnstone, and Wilson examined the robustness of SVM for predicting credit rating changes [32]. In the detection of stock market manipulation, Liu, Wang, Zhang, and Zheng directly utilized the SVM method to make robust prediction, analyzed the information from the CSRC punishment cases, and finally detected stock market manipulation in advance. In addition, by comparing the detection effectiveness of SVM and logistic regression, they found that SVM had stronger accuracy and robustness before and after balancing data [33]. At present, machine learning algorithms are less applied in the field of accounting information. According to existing literature, machine learning methods can effectively overcome nonlinear characteristics, and SVM is suitable for both structured and unstructured data [33]. As a result, we can introduce SVM algorithms to analyze environmental administrative penalty cases and accurately detect EIDF.

Class-imbalance deteriorates the performance of the classifier and affects the classification accuracy of the model. According to the existing literature, the methods for improving sample imbalance at the data level are more mature. Among the methods, oversampling and undersampling are two of the most widely used methods. Xie and Qiu believed that the method of oversampling was better than undersampling [34]; hence, in this study, we give priority to the oversampling method. And for the problem of distribution marginalization arising from the synthetic minority oversampling technique SMOTE [35], we further introduce the Borderline-SMOTE algorithm [12] to improve it by using only the smallest sample on the boundary to synthesize a new sample.

## 3. Methodology

Machine learning models for the detection of falsification are the core of this study. In this section, we first introduce the input feature metrics, followed by the theoretical structure of the models and the evaluation methods. In order to avoid the overfitting of the models in the training set and affecting the detection effect, we evaluate different models mainly according to the test results of the test set. In order to comprehensively evaluate the detection effect of falsification, we introduce the multidimensional evaluation indexes such as confusion matrix, accuracy, precision, recall, and F1 value and further introduce the receiver operating characteristic curve and area under curve. The details are shown below.

### 3.1. Constructing Eigenvectors

The outcome variables are mainly binary variables that indicate whether environmental disclosure falsification occurs, as shown in Table 1, and the basic input variables contain three main types of characteristics: public pressure, corporate governance, and financial indicators. In this section, we will explain the potential relationships between the selected characteristic variables and the outcome variables based on the motivation analysis of disclosure falsification behavior, and elaborate on the mechanism for the detection of EIDF behavior.

The first feature indicator is related to public pressure. The sources of public pressure on enterprises are mainly industry pressure, government pressure, investor pressure, local pressure, public opinion pressure, regulatory pressure, and social reputation. The industry type of enterprises is an important influencing factor of EID [36, 37]; for example, heavily polluting industries are more likely to pollute the environment and have always been the focus of regulation and inspection; therefore, they are inclined to face greater industry pressure. It is beyond cavil that all enterprises will face the pressure brought by government regulation, but enterprises of different ownerships face different government pressure. For instance, state-owned enterprises are more sensitive to government environmental regulation and tend to comprehensively improve the quality of their EID under regulatory pressure [38, 39]. For the pressure from investors, in order to gain their support, enterprises will increase the scope and intensity of EID to create a good image of environmental performance [40], and falsification is less likely to occur at this time. For local pressure, the high level of regional economic development can provide much more convenience for local enterprises to perform their environmental duty. When enterprises face greater local pressure, they tend to factually disclose environmental information [41]. For public opinion pressure, media exposure of enterprises' negative information will directly increase public pressure on themselves. Media pressure works as a deterrent role, prompts enterprises to abide by the law and regulations, fulfills their environmental responsibility, improves the level of EID [42], and restrains their falsification behavior. Regulatory pressure reflects the rigor and professionalism of the stock exchange. The greater the pressure, the more it can prompt information disclosure behavior to become legal and compliant [11], thus inhibiting the opportunistic behavior of enterprises. For the pressure from social reputation, quality system certification (ISO9001) reflects the quality reputation of the enterprises, and environmental management system certification (ISO14001) reflects the environmental management reputation. Many scholars believe that such voluntary quality certifications are effective in improving the enterprises' environmental performance. This effect is more significant after the promulgation and implementation of the new environmental protection law [43]. Especially for developing countries, this kind of quality certification can also promote the enterprises to comply with the requirements of the environmental law [44] and thereby inhibits EIDF.

At the corporate governance level, we selected internal supervision and equity structure as explanatory variables. First, we use the size of the corporate supervisory board to reflect the extent of internal supervision; the supervisory board can monitor the legal compliance of the enterprise's executives in performing their duties and expanding the listed enterprises' supervisory board is conducive to the functioning of EID system, therefore making it less prone to falsification [13]. Second, an unreasonable equity structure will give rise to more frequent occurrence of EIDF. On this account, we choose equity concentration and equity balances as the detection indicators. For one thing, the moderate increase of equity concentration is beneficial for the controlling shareholders to monitor the behavior of corporate management and safeguard their interests [45], thus restraining the occurrence of falsification. For another thing, the equity balance degree reflects the checks and balances of other shareholders against the top shareholders. Equity checks and balances can also reduce the enterprises' agency costs, effectively monitor managerial behavior, and maintain corporate value [46, 47], which will together have a positive effect on inhibiting falsification.

Finally, we combine financial indicators to reflect enterprises' motives for falsification. For the purpose of financing convenience and reducing financing costs, enterprises are more likely to falsify and whitewash environmental information when their performance declines and funds tighten [48]; therefore, a decline in profitability increases the probability of falsification. Secondly, rising liabilities will increase enterprises' pressure from creditors, which may lead them to reduce the disclosure of negative environmental information in order to alleviate creditors' sensitivity to environmental risks [49]. Thirdly, from the perspective of enterprise growth capacity, enterprises with ideal operating income growth rate and market capitalization size would have more reasonable capital structure, more sound internal management mechanism, higher level of EID [50–52], and less probability of falsification behavior.

### 3.2. Modeling Method

In this section, we introduce a SVM model for detecting environmental information falsification. The core idea of SVM is to solve for the separating hyperplane that correctly partitions the training data set and has the largest geometric separation. To demonstrate this algorithm, we assume that we have a training set of

(1) $$\begin{array}{c} Τ=\left\{\left(x_{1},y_{1}\right),\left(x_{2},y_{2}\right),...,\left(x_{n},y_{n}\right)\right\},\\ y_{i}\in \left\{-1,1\right\},\;i=1,2,...,N, \end{array}$$

where  *x*_*i*_ is the EID feature vector, as shown in indicator system construction, and *y*_*i*_ is the sample category, which means whether the environmental information *i* disclosed by the enterprise is falsified or not, and we define the falsified samples as positive samples and the not falsified samples as negative samples, and assume that the training set samples are linearly divisible and the hyperplane can be expressed as

(2) $$\begin{array}{c} \omega^{T}\cdot x+b=0, \end{array}$$

where *ω* is the adjustable weight variable and *b* is the bias vector.

In the actual use of SVM for the detection of counterfeiting behavior, it is not possible to find a completely linearly divisible dataset, so we need to introduce a relaxation variable *ξ*_*i*_, which is not less than 0 and a non-negative penalty factor *C*. In this case, the problem of solving the optimal classification hyperplane can be further expressed as

(3) $$\begin{array}{c} \min\frac{1}{2}\omega^{2}+C\sum_{i=1}^{n}\xi_{i},\\ \text{s.t}.\left\{\begin{array}{c} y_{i}\left(\omega^{T}x_{i}+b\right)\geq 1-\xi_{i},\;i=1,2,\ldots ,n\\ \xi_{i}\geq 0 \end{array}\right.. \end{array}$$

Considering that our samples are not simply linearly divisible, we need to introduce kernel functions to map linearly indivisible problems in two dimensions to a higher dimensional space. There are four types of kernel functions: linear kernel function, *d*-order polynomial kernel function, radial basis kernel function (RBF), and Sigmoid kernel function. These four types of kernel functions all contain adjustable parameters, and the combination of different kernel parameters and different penalty factors *C* will cause the SVM to exhibit different performance. Therefore, it is important to choose the appropriate kernel function parameters and penalty factor values in the process of utilizing SVM.

Meanwhile, we built a benchmark model, which is also a logistic regression model, and compared its results with the SVM model to verify the validity and robustness of the SVM model. The main idea of logistic regression is to add the sigmoid function to linear regression. By using the monotonicity of this function, the predicted value of linear regression can be transformed into a prediction about *P*{*Y*=1*|X*}, with a default threshold of 0.5. It is classified as one class when the value of *P* is greater than 0.5, under this circumstance, the value of *Y* has a higher probability of 1 (falsified). And it is classified as another class when *P* is less than 0.5, and the value of *Y* at this time has a higher probability of -1 (not falsified), so the logistic regression is able to deal with dichotomous problems. Based on this principle, let *p*=*p*(*y*_*i*_=1*|x*_*i*_) be the probability of EIDF of eigenvector *x*_*i*_, and according to the predictive function of logistic regression, it can be expressed as *p*=*p*(*y*_*i*_=1*|x*_*i*_)=1/1+*e*^−*θ*^*T*^*x*_*i*_^, where *θ* is the coefficient matrix, so the probability of not falsifying can be expressed as

(4) $$\begin{array}{c} 1-p=p\left(y_{i}=-1|x_{i}\right)=\frac{1}{1+e^{\theta^{T}x_{i}}}. \end{array}$$

### 3.3. Borderline-SMOTE

Considering that our sample dataset is unbalanced, we oversample the minority groups based on the Borderline-SMOTE algorithm proposed by Han et al. [12]. This algorithm takes into account the distribution of the samples and makes the synthetic minority class samples more targeted. Specifically, we assume that the minority class sample set in the training set S is *S*_min_ (falsified corporates), and the majority class sample set is *S*_maj_ (not falsified corporates). *T* and *Q* are used to represent the sample capacity, and then the minority group is oversampled by the following steps:

  Step 1: for each minority class sample, calculate *k*-nearest neighbors, and assume the number of majority class samples in the *k*-nearest neighbors is *k*′.
  Step 2: when *k* =  *k*′, the minority sample is judged as “noisy sample.” When 0 ≤*k*′ < *k*/2, the sample is judged as “safe sample”; when *k*/2 ≤ *k*′ < *k*, we take out the dangerous samples to participate in the next steps.
  Step 3: for each dangerous sample *x*_*i*_, we randomly select a sample(*x*_*i*_)from its nearest neighboring minority class *S*_min_ and take a random number *δ* from 0 to 1 to generate a new sample *x*_new_, where $x_{\text{new}}=x_{i}+\delta \cdot \left(\tilde{x_{i}}-x_{i}\right)$, thus obtaining a new minority class sample set *S*_min_', and this new minority class sample set and the original majority class sample set form a new training set sample *S*_new_ for model training.

### 3.4. Performance Evaluation

**Figure 1:**
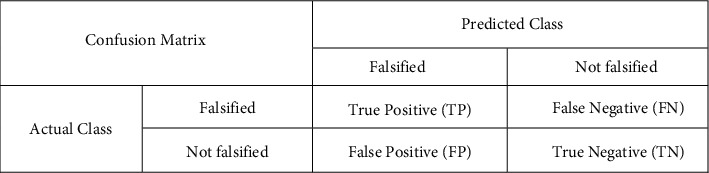
Two-category confusion matrix framework.

According to Kohavi and Provost [53], the confusion matrix contains information about the actual and predicted classifications performed by classification algorithms, and the data in the matrix are usually used to evaluate the performance of such algorithms. The framework of confusion matrix for binary classification is given in Figure 1.

On the basis of the confusion matrix, we further introduce multidimensional evaluation indicators as follows:

(5) $$\begin{array}{c} \text{Accuracy}=TP+\frac{TN}{\left(TP+TN+FP+FN\right)},\\ \text{Precision}=\frac{TP}{\left(TP+FP\right)},\\ \text{Recall}=\frac{TP}{\left(TP+FN\right)},\\ F1\text{Value}=2*\frac{TP}{\left(2*TP+FN+FP\right)}=\frac{2*\text{Recall}+\text{Precision}}{\left(\text{Recall}+\text{Precision}\right)}. \end{array}$$

The accuracy rate represents the ratio of all correctly predicted samples to the total samples, and generally speaking, the closer to 1, the better. However, when the dataset is unbalanced, the classifier will easily be inclined to the majority class [54], and when the accuracy will not be a good representation of the model's performance, and there may be cases where the accuracy is high and the minority class samples are all wrongly scored [55], other model evaluation metrics should be selected.

The precision rate indicates the proportion of all samples that we predict as falsified that are truly falsified. A higher precision rate indicates a more accurate prediction for a small number of classes, and in most cases, the precision rate decreases after the samples are balanced. The recall rate indicates the proportion of all truly falsified samples that are correctly predicted by us, and a higher value indicates that we try to catch as many falsification samples as possible. F1 value is the harmonic mean of accuracy rate and recall rate, which reflects the robustness of the model. Its value is distributed between [0,1], and the closer to 1 the better the model is evaluated.

#### 3.4.1. ROC Curve and AUC

ROC curve refers to the receiver operating characteristic curve, which is a curve with the false-positive case rate FPR at different thresholds as the horizontal coordinate and the true case rate TPR at different thresholds as the vertical coordinate [33]. It reveals the interrelationship between sensitivity and specificity by using the composition method, where TPR = TP/(TP + FN), which indicates the proportion of the predicted category that is falsified among all samples whose true category is falsified; and FPR = FP/(FP + TN), which indicates the proportion of the predicted category that is not falsified among all samples whose true category is not falsified [56]. By taking the values of the classification threshold (default is 0.5) from large to small or from small to large in order, we can get many sets of TPR and FPR values, and a ROC curve can be obtained by drawing them in order in the image.

**Figure 2:**
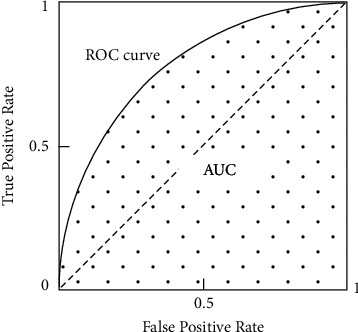
ROC curve and AUC. (Source of the picture: Liu et al. [33]).

The AUC value represents the area enclosed by the ROC curve with the horizontal axis and the straight line FPR = 1, as shown in Figure 2. AUC can be used as a metric to evaluate model performance, but it can only be used for the evaluation of binary classification models. Through comparing different ways of model evaluation, Jin and Ling concluded that AUC is a better metric than accuracy [57]. For the data with category imbalance, the AUC calculation method can still make a reasonable evaluation of the classifier. Usually, when AUC > 0.5, it indicates that the classifier is effective. And the larger the value, the better the classification of the model will be. When AUC = 1, it is called a perfect classifier, but in most cases, there is no perfect classifier.

## 4. Data

We systematically identify falsification cases from the search of the Public Environmental Research Center (IPE) and GreenNet Environmental Data Center by using environmental administrative punishment cases of nonfinancial listed enterprises in China's A-share market from 2015 to 2019 and manually collect a total of 704 punishment records. In terms of the time span of the punishment cases, we firstly collected data for a ten-year period from 2010 to 2019. However, China has issued the new Environmental Protection Law in 2015, since when the government and regulatory authorities have imposed more stringent requirements on corporate EID and clearly stipulated that listed companies should actively disclose environmental information. Although the level of corporate EID has been greatly improved, the falsification behavior has also become more insidious than before. In addition, COVID-19 has had abnormal and extreme negative impacts on the business environment for the vast majority of enterprises. And our empirical research must be based on small sample data from normal years, based on SVM to solve the detection problem of EIDF. The advantage of SVM is its application to intelligent learning problems in combination with function fitting with the help of small sample data. Therefore, we did not use data from 2020 and beyond. Taking those factors into consideration, we choose the data from 2015 to 2019 as the sample period for our study.

We processed all the indicators of listed enterprises in nonfinancial industries, excluding enterprises that have been delisted and those with missing data, and matched them with enterprises with penalty records. Taking the year as the unit, 14,856 samples are finally obtained, including 529 positive samples involving 183 companies that have been falsified and 14,327 negative samples that have not been falsified. The data of other variables are obtained from CSMAR database, RESSET database, Zhonghong Economic Research and Application Platform, and China Research Data Service Platform (CNRDS).

In the modeling process, we set the year as a dummy variable to separate the influence of anomalous factors in different years to make our data comparable. Meanwhile, since the falsified sample only accounts for 3.56% of the total sample, we use the Borderline-SMOTE algorithm described in Section 3.2 to deal with sample category imbalance. In order to obtain more reliable results, this study takes 80% of the dataset as the training set to construct the detection model of EIDF. The remaining 20% is used as the test set in the model testing to evaluate whether the model is effective.

In order to characterize the falsified samples, we compared the data from positive and negative samples.  Table 2 gives a comparison of the mean value of the positive and negative samples. Preliminary conclusions can be drawn from the table: first of all, in terms of public pressure, enterprises in heavy polluting industries are more likely to falsify EID, and they will face greater pressure from industry, government, and social reputation. However, the greater the investor pressure, local pressure, public opinion pressure, and regulatory pressure, the less likely the falsification is to occur. Secondly, in terms of corporate governance, the larger the size of the supervisory board and the higher the ownership concentration, the lower the probability of falsification will be. Finally, in terms of financial indicators, enterprises with poor profitability, high gearing ratio, and low growth capacity have a higher probability of falsification. The results of the above descriptive tests preliminarily verify the logic of the detection of falsification in EID that is described in indicator system construction.

## 5. Experimental Results and Analysis

### 5.1. Model Detection Operation

In this section, we first apply machine learning logistic regression and support vector machine algorithms to detect environmental disclosure falsification on the original dataset, without considering the sample class imbalance. As mentioned earlier, logistic regression is a classical and popular machine learning algorithm, which is simple to operate, easy to understand, and fast to run. However, our dataset is not simply linearly separable, and we believe a nonlinear SVM with kernel functions may have better detection results, so we will compare and analyze the detection results of the two models. In this study, we use Python software to realize the operation of the model.

We used 80% of the randomly selected samples as the training set to get 11884 samples. The optimal parameter settings of the model are obtained by using fivefold cross-validation, making the model training results more accurate. The training set detection results are shown in Table 3. And the remaining 2972 samples are used as the test set to verify the model's detection recognition ability, and the test results are shown in Table 4. Since the test set detection results may be overfitted or affected by other reasons, more attention will be focused on the results of the test set.

Table 4 shows the model detection performance of the two algorithms, logistic regression, and SVM, without considering whether the data are balanced or not. From the performance evaluation panel, we can see that both SVM and logistic have relatively high accuracy, and SVM has a higher accuracy of 96.51%. However, through comprehensive analysis of the confusion matrix, we find that the high accuracy results are for majority samples, that is, the nonfalsified samples, and most of them are correctly classified. Yet the classification for minority samples is wrong. At this point, the accuracy rate does not represent the performance of the model well, and we need to further introduce the multidimensional evaluation index *F*1 value and AUC value. For the two algorithms, if the *F*1 value is 0 and the AUC value is 0.5, it indicates the effect of logistic regression and SVM algorithms is equivalent to a casual guess in the case of data imbalance, and the model has no predictive value. Therefore, in order to build an effective model for detecting environmental disclosure falsification, we use Borderline-SMOTE to balance the two types of samples in the next section.

### 5.2. Detection Operation after Handling Data Imbalance

In this section, we further use the oversampling method Borderline-SMOTE to deal with the data imbalance between falsified and nonoperated samples based on the previous subsection. We construct the falsification detection models Borderline-SMOTE-SVM and Borderline-SMOTE-Logistic, and show the training set sample detection results of both models in Table 5.

Table 6 shows the detection results of the test set after dealing with the data imbalance by the Borderline-SMOTE method. It can be seen from Table 6 that the performance of both classifiers is significantly improved after utilizing the Borderline-SMOTE method. Compared with the results in the previous section, the recall rate increased the most, from 0 to 68.27% (SVM) and 66.35% (logistic), indicating that our model captures as many falsified samples as possible after the oversampling, and the accuracy rate still remains around 75%, indicating that the model is still accurate in detecting falsifications. For the accuracy rate and *F*1 values, although the results have improved from the previous section, they are not very satisfactory overall. Therefore, we further consider the AUC values to assess the model effects.

**Figure 3:**
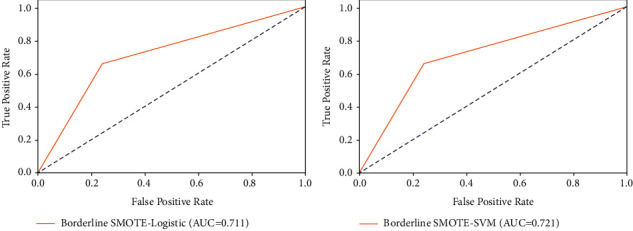
Working characteristic curves of the test set receivers of the model. *Note.* ROC curves and AUC values for Borderline-SMOTE-logistic, Borderline-SMOTE-SVM.

Figure 3 shows the ROC curves and AUC values of Borderline-SMOTE-SVM and Borderline-SMOTE-Logistic, and we can see that the AUC values increase to 0.721 and 0.711, indicating that the model has better detection effect after oversampling. And both the traditional logistic regression model and the nonlinear SVM model are effective in detecting the falsification of EID. From the AUC value, SVM has more advantages than logistic.

### 5.3. Robustness Testing

**Figure 4:**
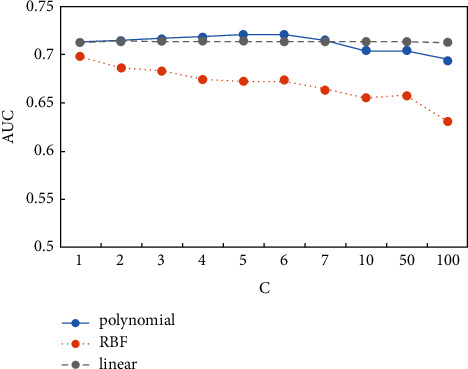
Stability test of the SVM model based on Borderline-SMOTE.

For machine learning algorithms, adjusting the default parameter settings will have a crucial impact on the performance of the model, and the model detection results will also change. When using Python software to train our SVM model, the choice of kernel function and hyperparameter is the core setting for us. Therefore, to further verify the robustness of the model, we select three kernel functions (linear, RBF and polynomial) and combined them with different penalty coefficients (C) to observe the AUC values under different kernel functions and parameter. The results are shown in Figure 4. Before training, we balance the data based on the Borderline-SMOTE algorithm and find the optimal settings of other parameters by five times of cross-validation.

Firstly, the penalty coefficient indicates the tolerance of the model to errors. The higher the C, the higher the penalty and the less tolerance for errors. Therefore, the classification will be more accurate but prone to overfitting, while smaller C might lead to underfitting. Whether C is too large or too small, it will both make the generalization ability poor. As is shown in Figure 4, for the same kernel function, the variation of AUC value is small under 10 sets of different penalty coefficients, and its variation range is maintained within 0.1.

Secondly, the values of AUC are kept between 0.63 and 0.721 under different kernel functions. And when polynomial kernel function is selected and penalty coefficient is 6, AUC reaches the maximum value of 0.721. In general, the polynomial kernel function performs best with an AUC value of about 0.72, followed by the linear kernel function with an AUC value of about 0.713 and the radial basis kernel function with an AUC value of about 0.68.

In summary, it can be seen that Borderline-SMOTE-SVM has good detection ability with the AUC values greater than 0.63 under the two parameter settings of kernel function and penalty coefficient (C). And by setting the combination of parameters, it can make the AUC value reach 0.721, which is better than logistic regression model of 0.711. Therefore, it can be assumed that the Borderline-SMOTE-SVM model has strong robustness.

## 6. Conclusion

In the implementation of Carbon Peak and Carbon Neutrality strategy, enterprises are facing increasing pressure from environmental protection, and the behavior of EIDF tends to be more secretive. It may create a false environment-friendly image, resulting in a higher degree of information asymmetry, and thus, damaging the interests of investors and disrupting the market regulatory order. It may even cause environmental deterioration and ecological disorder, and hinder the green development. With the awakening of social public environmental awareness, EID is considered a key to corporate green governance. Therefore, how to effectively detect EIDF is the focus of supervision.

This study establishes a machine learning model for detecting EIDF based on the manually collected environmental administrative penalty cases and compares the model with the basic algorithm logistic regression. Meanwhile, Borderline-SMOTE oversampling technique is introduced to deal with data imbalance. In addition, we construct a feature vector set including public pressure, corporate governance, and financial indicators. We find that the Borderline-SMOTE algorithm can improve the effectiveness of model detection with an AUC value of more than 70%, and the test set detection performance of the support vector machine under this algorithm outperforms that of the basic logistic regression model. Finally, we carry out a robustness test, and the performance of our model testing is consistent under different parameter settings.

This study will be of practical significance to regulatory agencies, because Borderline-SMOTE-SVM can effectively detect corporate EIDF and thus play a warning role in regulation.

For investors, our EIDF detection index system can help them to be more alert to the environmental information disclosed by heavily polluting enterprises with poor profitability, high gearing ratio, and small supervisory boards, so as to avoid losses caused by information asymmetry.

For the third-party service sector like financial institutions, our evaluation system and detection method can help them assess the transparency of corporate EID, so as to identify and manage environmental-related financial risks, promoting the sound operation of the financial system simultaneously.

For enterprises, they should improve their corporate governance structure and actively turn environmental pressure into a driving force for innovation, transformation, and upgradation of development modes and also promote sustainable economic development and green competitiveness.

For government policy-making, it is necessary to improve the formulation of EID laws and regulations and establish the EID criterion and standard system. The government should require enterprises to disclose not only the performance on social and environmental issues, but also their future improvements. Disclosure priorities have become the government's dominant policy to improve the level of transparency.

There are still some limitations in this study. Firstly, our detection indicators of EIDF are based on the motivation logic of “public pressure.” In fact, the motivation for falsification is rather complex, and there should be other factors. For example, on objective factors, enterprises may be influenced by market sentiment and the market's intensity of competition. On subjective factors, corporate transparency and corporate culture [58] will also affect the authenticity of EID. How to improve the data availability and index system is worthy of further study. Secondly, it is worth exploring how to improve the machine learning technology and the algorithm of unbalanced data processing to make EIDF detecting model more accurate and reliable. Thirdly, we mainly focus on enterprises that have already committed EIDF, and how to provide early warning of EIDF will be a direction for future research. Finally, we do not measure weight to different industries. How to quantify industrial characteristics and measure them specific weights will be valuable research directions in the future.

## Figures and Tables

**Table 1:** Final inputs for detecting falsification in machine learning models.

		If falsification of environmental information occurs, the value is 1; otherwise, it is −1		
		Projects	Specific variable name	Variable measurement
Explanatory variables	Public pressure	Industry pressure	Nature of industry	Whether the enterprise belongs to the heavy polluting industry: if yes, then 1; otherwise, 0
		Government pressure	Nature of property rights	State-owned and state-owned holding company takes 1, otherwise takes 0
		Investor pressure	Earnings per share	Ratio of profit after tax to total share capital
		Local pressure	Regional economic development level	GDP per capita value of the province where the company is incorporated
		Public opinion pressure	Public opinion index	Natural logarithm of the annual number of media reports on the company
		Regulatory pressure	Exchange regulation	1 if the company is listed on the SSE, 0 otherwise
		Social reputation	ISO9001 certification	1 if certified, 0 otherwise
			ISO14001 certification	1 if certified, 0 otherwise
	Corporate governance	Internal supervision	Supervisory board size	Number of supervisory board, a standing body that performs supervisory authority
		Shareholding structure	Concentration of shareholding	Share of the share of the first largest shareholder in the total shares of the company
			Shareholding checks and balances	Ratio of the sum of the shareholding shares of the second to tenth largest shareholders to the shareholding share of the first largest shareholder
	Financial indicators	Profitability	Net profit ratio of total assets	Net profit/average balance of total assets
		Solvency	Asset debt ratio	Total liabilities/total assets
		Growth capacity	Operating income growth rate	(Current year-previous year's gross operating income)/previous year's gross operating income
			Company size	Natural logarithm of total assets of listed enterprises at the beginning of the year

**Table 2:** Summary statistics of mean values of all variables.

		Variables	Falsification sample mean	Not falsification sample mean
	Result variables	Fraudulent	1	−1
Public pressure	Industry pressure	Nature of industry	0.638941399	0.251553012
	Government pressure	Nature of property rights	0.351606805	0.332239827
	Investor pressure	Earnings per share	0.204201989	0.342566624
	Local pressure	Regional economic development level	73088.40265	83214.80233
	Public opinion pressure	Public opinion index	5.059937048	5.079496133
	Regulatory pressure	Exchange regulation	0.323251418	0.385844908
	Social reputation	ISO9001 certification	0.27221172	0.234661827
		ISO14001 certification	0.285444234	0.225169261
Corporate governance	Internal supervision	Supervisory board size	3.482041588	3.48984435
	Shareholding structure	Concentration of shareholding	32.78454159	33.40385589
		Shareholding checks and balances	0.911224741	1.00977148
Financial indicators	Profitability	Net profit margin on total assets	0.031293868	0.033354032
	Solvency	Gearing ratio	0.44887276	0.432038349
	Growth capacity	Operating income growth rate	0.170986217	0.510638997
		Company size	22.09202674	22.16087934

**Table 3:** Model training set detection results.

	SVM	Logistic regression
Accuracy	0.9643	0.9642
AUC value	0.5000	0.5000

**Table 4:** Test set sample detection results.

*A. Logistic regression confusion matrix*		
	Forecast falsification	Forecast not falsified
Actual falsification	0	104
Actual not falsified	1	2867

*B. SVM confusion matrix*		
	Forecast falsification	Forecast not falsified
Actual falsification	0	104
Actual not falsified	0	2868

*C. Performance evaluation*		
	SVM	Logistic regression
Accuracy rate	0.9651	0.9648
Accuracy rate	0	0
Recall rate	0	0
F1 value	0	0
AUC value	0.5000	0.5000

**Table 5:** Model training set detection results.

	SVM	Logistic regression
Detection results of training set using Borderline-SMOTE processing data		
Accuracy	0.7870	0.7630
AUC value	0.6960	0.6860

**Table 6:** Test set sample detection results after processing with Borderline-SMOTE.

*A. Logistic regression confusion matrix*		
	Forecast falsification	Forecast not falsified
Actual falsification	69	35
Actual not falsified	692	2176

*B. Support vector machine confusion matrix*		
	Forecast falsification	Forecast not falsified
Actual falsification	71	33
Actual not falsified	689	2179

*C. Performance evaluation*		
	Borderline-SMOTE-SVM	Borderline-SMOTE-logistic
Accuracy rate	0.7571	0.7554
Accuracy rate	0.0934	0.0907
Recall rate	0.6827	0.6635
*F*1 value	0.1644	0.1595
AUC value	0.7210	0.7110

## Data Availability

The data used to support the findings of this study are available from the corresponding author upon request.
